# Effects of changes in polycyclic aromatic hydrocarbons (PAHs) emissions and degradation on their concentrations in Tokyo from 2007 and 2016

**DOI:** 10.1038/s41598-022-08138-8

**Published:** 2022-03-11

**Authors:** Kojiro Shimada, Masayuki Nohchi, Koji Maeshima, Tomonori Uchino, Yusuke Kobayashi, Kazuki Ono, Hiroko Ogata, Naoya Katsumi, Koji Inazu, Hiroshi Okochi

**Affiliations:** 1 School of Creative Science and Engineering, Waseda University, 3-4-1 Okubo, Shinjuku, Tokyo 169-8555 Japan; 2 Department of Bioresources and Environmental Sciences, Ishikawa Prefectural University, 1-308 Suematsu, Nonoichi, Ishikawa 921-8836 Japan; 3 National Institute of Technology, Numazu College, 3600 Ooka, Numazu City, Shizuoka 410-8501 Japan; 4 Present Address: Department of Chemistry, Biology, and Marine Science, University of the Ryukyus, Okinawa, 903-0213 Japan

**Keywords:** Environmental sciences, Risk factors

## Abstract

The concentrations of polycyclic aromatic hydrocarbons (PAHs) in aerosol were measured in Shinjuku, which is central Tokyo, Japan, for 10 years from 2007 to 2016. The effects of changes in emission sources and their degradation by reaction with ozone were assessed in this study. There was no significant increasing or decreasing trend of the PAH concentrations during 10 years (*P* > 0.05). The average selected seven the PAH concentrations (0.88 ng m^−3^) during 10 years was lower than those in New York and Paris. However, the trend of ozone concentrations is increasing in central Tokyo. This inconsistency raises a question. Did the fact that the ozone concentration was higher than the PAH concentrations promote PAH degradation? To apportion the PAH sources, we used PAH concentration profiles and positive matrix factorization analysis. The contribution of vehicle emissions to the PAHs ranged from 40 to 80%. Ozone concentrations increased by 3.70%/year during 10 years. The theoretical degradation rates of PAHs by ozone, which were calculated using a pseudo-first-order rate equation, suggested that the lifetimes of benzo[*a*]pyrene (BaP) decreased by 1 min from 2007 to 2016. We investigated the aging of BaP using the profile of the isomer ratios. We found that the aging of BaP at the urban and roadside sites were nearly identical indicating aging regardless of the season. Although the decomposition of BaP is promoted by the photochemical oxidation reaction, this result suggests that a certain threshold value exists as the degree of the decomposition. This degradation of PAH can improve chemical loss processes in air quality model.

Polycyclic aromatic hydrocarbons (PAHs) are carcinogenic and/or mutagenic chemical component in PM_2.5_^1^. The International Agency for Research on Cancer assigns benzo[*a*]pyrene (BaP) to Group 1 (carcinogenic to humans)^2^.

Recently, emission of air pollutants controls has been implemented based on the national ambient air quality standards (NAAQSs) in representative megacities such as Japan, the United States and Europe^3–5^. One countermeasure against air pollution is regulation of emissions from diesel vehicles^6,7^. As a result, the concentrations of air pollutants such as NO_*x*_ and PM_2.5_ in ambient air at roadside sites slightly have decreased^7,8^. On the other hand, although the trend of ozone concentrations is decreasing in the United States and Europe, the trend is increasing at central Tokyo in Japan^9^. Titration of ozone by NO is low because of decreased NO_*x*_ concentration. Consequently, the trend of ozone concentrations is increasing in central Tokyo^10^. As above, the ozone concentrations in central Tokyo has characteristics trend compared to other countries such as New York and Paris, which are representative megacities in the United States and Europe where emission controls have been implemented.

PAHs are rapidly degraded by ozone^11,12^. Heterogeneous reaction of PAHs with ozone is faster than the photochemical reactions^13,14^. Long-term trends of atmospheric PAH concentrations can be expected further the decreased PAH concentrations in central Tokyo because high ozone concentration can promote PAH degradation.

We observed PAH concentrations for 10 years (2007–2016) in central Tokyo (Shinjuku).

Herein, we describe our results on the change of long-term trends of PAH concentrations in central Tokyo. In particular, we investigated the long-term trends of BaP degradation with increasing ozone concentration in central Tokyo.

## Results and discussion

### Annual variation of PAH concentrations (2007–2016)

**Figure 1:**
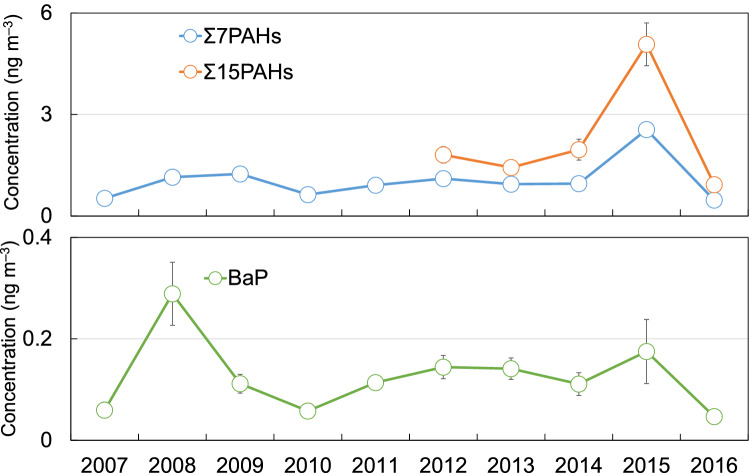
Average annual ΣPAH and BaP concentrations, 2007–2016.

In Shinjuku, measurements were conducted during 40 weeks in four seasons from 2007 to 2016, and a total of 436 samples were collected. The annual average Σ7PAH and Σ15PAH concentrations, which are defined as the sum of seven PAHs concentration and fifteen PAHs concentration, respectively, are shown in Fig. 1. Detailed information about the observation periods and the numbers of samples is shown in Table 1. The Mann–Kendall trend test^15^ showed no significant increasing or decreasing trends (*P* > 0.05) of the PAH concentration data over the course of the study period, although the average Σ7PAH and Σ15PAH concentrations spiked in 2015; suggesting that, there was little change in the PAH concentrations over the course of the 10 years from 2007.

**Table 1:** Sampling periods, numbers of samples, and Σ7PAH and Σ15PAH concentrations, 2007–2016.

Observation period		No. of samples	Σ7PAHs (ng m^−3^)	Σ15PAHs (ng m^−3^)
2007	8/1–8/7	14	0.521	ND
2008	7/22–7/29	15	1.15	ND
2009	2/23–2/28	10	1.95	ND
	5/26–5/31	9	0.521	ND
	7/17–7/27	22	1.04	ND
	10/19–10/27	15	1.09	ND
2010	5/24–5/28	10	0.514	ND
	7/12–7/16	9	0.240	ND
	10/18–10/22	10	0.554	ND
	12/6–12/13	11	0.924	ND
2011	3/7–3/11	9	0.922	ND
	5/16–5/20	10	0.754	ND
	7/11–7/22	20	0.664	ND
	10/3–10/7	8	1.03	ND
	12/12–12/16	10	1.44	ND
2012	3/19–3/23	8	1.72	2.76
	5/14–5/18	9	1.25	1.88
	7/12–7/19	14	1.42	2.16
	8/17–8/24	12	0.773	1.28
	10/22–10/26	9	0.397	0.73
	12/3–12/7	10	0.889	1.53
2013	3/18–3/22	10	0.982	1.46
	5/13–5/17	9	1.29	1.97
	7/29–8/2	10	1.27	2.07
	8/15–8/22	13	0.481	0.761
	10/7–10/11	10	0.274	0.545
	12/2–12/6	9	1.89	2.69
2014	5/12–5/16	10	0.968	1.96
	7/13–7/19	13	0.899	1.76
	8/21–8/25	9	0.214	0.496
	10/20–10/24	10	1.43	2.75
	12/1–12/5	10	1.55	3.49
2015	4/6–4/10	10	1.17	1.90
	7/15–7/23	17	2.90	5.42
	8/18–8/21	6	3.11	6.29
	10/26–10/30	12	2.99	5.93
	11/30–12/4	10	2.44	6.24
2016	4/4–4/8	9	0.428	0.872
	7/12–7/15	7	0.390	0.629
	8/17–8/21	8	0.399	0.641

*ND* no data.

We compared the PAH concentrations in Shinjuku with them at megacities in the United States and Europe, where emission controls have been implemented. The average Σ8PAH concentration in New York City between 2001 and 2012 was 2.0 ng m^−3^^16^, and the PAH concentration measured in Paris since 2009–2010 using a photoelectric aerosol sensor was 4.6 ng m^−3^ in summer and 13.8 ng m^−3^ in winter^17^. The Σ7PAH and Σ15PAH concentrations were 0.88 ng m^−3^ and 1.5 ng m^−3^, respectively, and both of them were lower than those in New York and Paris. In addition, we compared the overall average BaP concentrations in Shinjuku with NAAQSs for BaP (Fig. 1). There are no environmental standards for PAHs in Japan. However, in Europe, NAAQSs for PAHs are defined. In Europe, the standard for annual average BaP concentration is 1 ng m^−3^^4^. In United Kingdom, national air quality objective for annual average BaP concentration is 0.25 ng m^−3^^18^. The overall average BaP concentrations measured in our study (0.05–0.29 ng m^−3^) were also lower than the standard for annual average BaP concentration (1 ng m^−3^) in Europe (Fig. 1).

**Figure 2:**
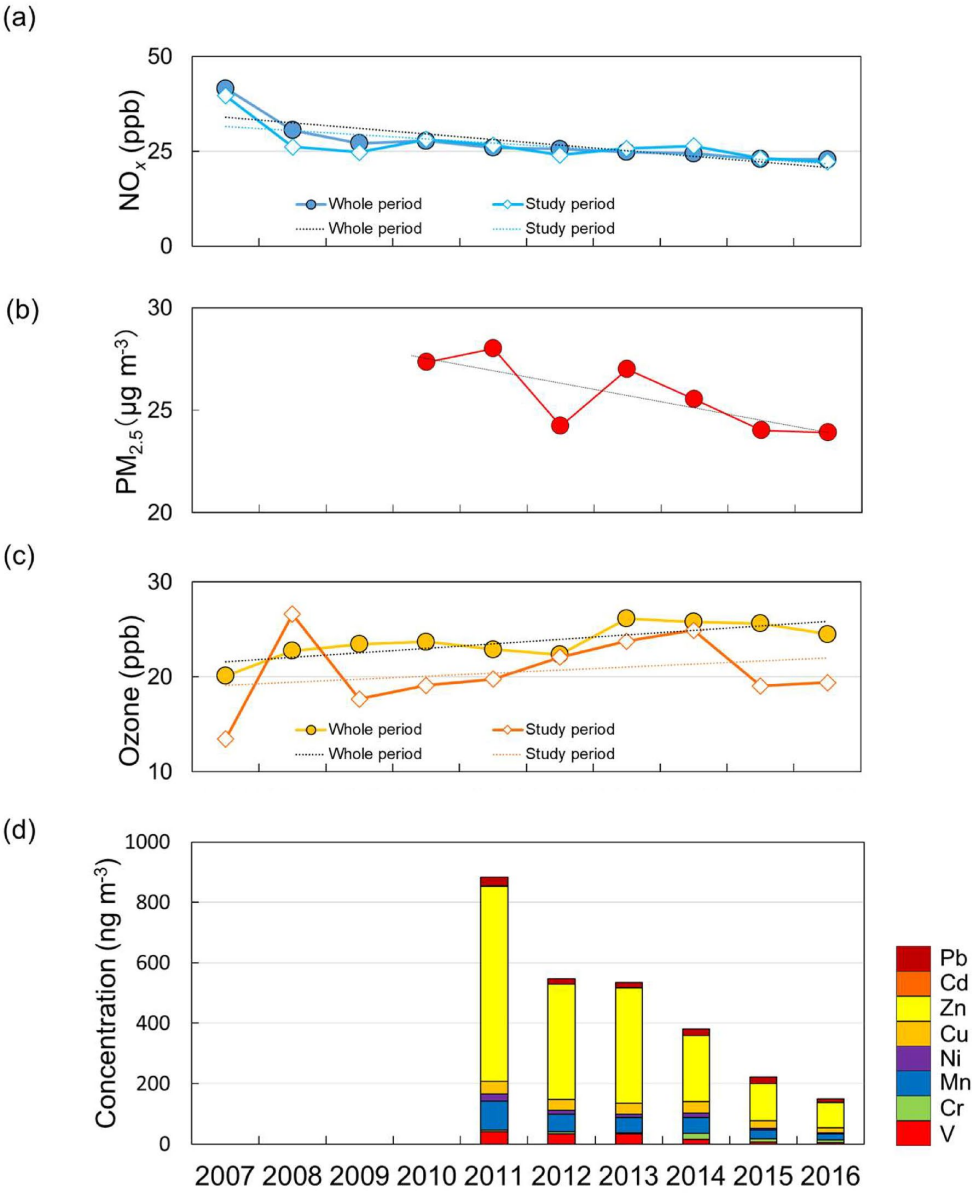
Average annual concentrations of (**a**) NO_*x*_ during 10 years (y =  − 1.48x + 35.6, R = 0.815) and only for the PAHs sampling period (y =  − 1.09x + 32.7, R = 0.68), (**b**) PM_2.5_ during the PAHs sampling period (y =  − 0.61x + 30.0, R = 0.76), (**c**) ozone during 10 years (y = 0.47x + 21.1, R = 0.77) and only for the PAHs sampling period (y = 0.72x + 15.7, R = 0.62 except for 2008) (**d**) anthropogenic elements during the PAHs sampling period, 2007–2016. NO_*x*_ and ozone data were collected hourly for 10 years and we extracted data for the PAHs sampling period described in Table 1 from the whole data sets. For more details, see “Methods” section in Supplemental data.

Figure 2 show the long-term trends of the concentrations of anthropogenic trace elements, namely Pd, Cd, Zn, Cu, Ni, Mn, Cr, and V in suspended particulate matter (SPM: *Dp* < 10 µm 100% cut point using impactor. For more details, see “Methods” in “Air sample collection” sections) with NO_*x*_, ozone, and PM_2.5_ concentration at the observation points closest to our sampling site. Unlike the PAH concentrations, the concentrations of anthropogenic trace elements, PM_2.5_, and NO_*x*_ tended to decrease from 2011 to 2016, at a rate of 25.3%/year for the anthropogenic trace elements (relative to the year 2011) and at rates of 3.87%/year for PM_2.5_ in this study period (relative to 2010) and 5.80%/year and 5.63%/year for NO_*x*_ in the 10 years whole and this study period (relative to 2007). The decrease in NO_*x*_ concentration is consistent with the long-term trend of NO_*x*_ concentration in the Kanto region, as indicated by analysis of results obtained using the Community Multiscale Air Quality model and NO_*x*_ observed from the satellite-based Ozone Monitoring Instrument^19^. These results reflect the effects of governmental measures aimed at reducing vehicle emissions^20^.

On the other hand, ozone concentrations increased gradually from 2007 to 2016 (2.00%/year and 3.70%/year in the 10 years whole period and overall period of this study: OP) (Fig. 2), due to VOC limited in central Tokyo^10^ is considered as well as the World Meteorological Organization (WMO) report^9^. Although the trend of ozone concentrations is decreasing in North America and Europe, the trend is increasing in East Asia including at central Tokyo in Japan, in particular^9^. Because ozone is consumed by conversion of vehicle-discharged NO to NO_2_ (Titration of ozone by NO), in particular, recent reductions in NO_*x*_ emissions in central Tokyo can be expected to have contributed to the increase in ozone concentrations^9,10^. In our study, the trend of ozone concentrations also shows the same result based on the report of WMO and Kanaya et al.^9,10^. However, the time series of PAH concentrations was not consistent with those of other air pollutants in this study.

This inconsistency raises several questions. Did the PAHs originate from a source other than vehicles? Did the fact that the ozone concentration was higher than the PAH concentrations promote PAH degradation? These two questions are discussed below.

### Source apportionment

To determine whether the PAHs originated from a source other than vehicles, we used (1) PAH concentration profiles and (2) PMF analysis to apportion the PAH sources. (1) PAHs concentration profile to identify source factors in PMF analysis, which are described in detail in the Supplement.

### PMF analysis to apportion the PAH sources

We used the following three datasets for PMF analysis: 15PAHs, 7PAHs, and PAHs + elements. To investigate the long-term trend of the contribution ratio to each PAH sources during 10 years from 2007 to 2016, we used the concentrations of 7 PAHs. However, we suspected that identifying PAH sources by means of PMF might be difficult because of the lack of information on sources of each PAHs. In order to use the dataset of 7 PAHs, we began by investigating the common factors in PMF by comparing the results for the dataset of 15 PAHs with the results for PAHs + elements. Moreover, using the procedure described by Han et al.^21^, we compared the source profiles of the factors identified with the 15 PAHs dataset and the factors identified with the dataset of PAHs + elements, either by inputting all the data for a given year as a single file or by inputting the data for each observation period as a separate file, with the goal of understanding the uncertainty of the PMF results. Details regarding the identification of the factors are described in the Supplement.

Here, we verify whether 7 PAHs dataset could be used to investigate the long-term trend of the contribution to each PAH sources by comparing results of 15 PAHs dataset based on the common factors identified using PMF analysis.

We carried out a PMF analysis with 15 PAHs dataset in SPM collected from 2012 to 2016. This 15 PAH analysis identified five factors (Fig. S4). The dataset of 7 PAHs collected from 2007 to 2016 were used in the PMF analysis, which identified four factors (Fig. S8).

**Figure 3:**
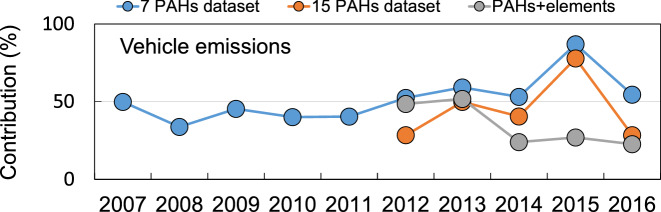
Contributions of vehicle emissions to 7 PAHs dataset, 15 PAHs dataset, and PAHs + elements, 2007–2016.

We compared gasoline vehicle emissions, diesel vehicle emissions, heavy oil combustion, and waste incineration, which were identified as sources shared in common by the datasets of 15 PAHs and the 7 PAHs. The factor profiles for the 7 PAHs dataset were consistent with the profiles for the 15 PAHs dataset. Hence, we found that the behaviors of both dataset before 2012 were consistent with each other. To better understand the long-term trend of the PAH concentrations, we evaluated the trends of the contributions of the major sources. Figure 3 shows variations of the contributions of vehicle emissions (gasoline + diesel) for the datasets of 7 PAHs, 15 PAHs, and PAHs + elements. Using the datasets of 7 PAHs and 15 PAHs, vehicle emissions accounted for 40–80% of the total PAHs. Whereas vehicle emissions contributed 20–50% using the dataset of PAHs + elements. The contributions of vehicle emissions using the datasets of PAHs + elements and 15 PAHs were similar from 2012 to 2016, except for in 2015. The contribution of vehicle emissions showed no seasonal variation (Fig. S10).

Therefore, the PAH concentration profiles and the PMF analysis indicated that the PAHs observed from 2007 to 2016 originated mainly from vehicles. However, the long-term trend of the PAH concentrations was affected by changes in the contributions of decreased vehicle emissions (Fig. 2).

### Degradation of PAHs with ozone

PAHs are rapidly degraded by photochemical reactions^14^. Heterogeneous reaction of PAHs with ozone is faster than the photochemical reactions^13,14^. It was necessary to investigate the effect of ozone concentration on PAH degradation. Because ozone concentration can be regarded as a constant and can be included in an apparent PAH decay rate coefficient, the degradation rate of PAH by ozone can be expressed by a pseudo-first-order rate equation under constant temperature conditions^11,22^:

1 $$\frac{{{\text{d}}[{\text{PAH}}]}}{{{\text{dt}}}} = - {\text{k}}^{{\prime}}_{1} [{\text{PAH}}]$$

where $[{\text{PAH}}]$ is the concentration of a given PAH, and ${\text{k}}^{\prime}_{1}$ is the pseudo-first-order rate constant. Rate constant ${\text{k}}^{\prime}_{1}$ depends strictly on ozone concentration:

2 $${\text{k}}^{\prime}_{1} = {\text{k}}_{2} [{\text{O}}_{3} ]$$

where ${\text{k}}_{2}$ is a second-order rate constant. The value of ${\text{k}}^{\prime}_{1}$ can be determined from the following linear equation:

3 $$\ln \frac{{[{\text{PAH}}]}}{{[{\text{PAH}}]_{0} }} = - {\text{k}}^{\prime}_{1} {\text{t}}$$

We investigated the relationship between ${\text{k}}^{\prime}_{1}$ and ozone concentration. At low ozone concentrations (< 5 × 10^12^ cm^−3^, ~ 200 ppb), ${\text{k}}^{\prime}_{1}$ increases almost linearly with increasing $[{\text{O}}_{3} ]$, but at higher ozone concentrations, ${\text{k}}^{\prime}_{1}$ becomes saturated at a value designated as ${\text{k}}^{\prime}_{\max }$^11^. This behavior is a feature of the Langmuir–Hinshelwood mechanism, which can be expressed in terms of the Pöschl–Rudich–Ammann model^23^:

4 $${\text{k}}^{\prime}_{1} = {\text{k}}^{\prime}_{{{\text{max}}}} \frac{{{\text{K}}_{{{\text{O}}_{3} }} [{\text{O}}_{3} ]}}{{1 + {\text{K}}_{{{\text{O}}_{3} }} [{\text{O}}_{3} ]}}$$

where ${\text{K}}_{{{\text{O}}_{3} }}$ is the ozone-gas-to-surface equilibrium constant, and $[{\text{O}}_{3} ]$ is the gas-phase ozone concentration. Pöschl et al.^11^ reported that the ${\text{k}}^{\prime}_{\max }$ and ${\text{K}}_{{{\text{O}}_{3} }}$ values for heterogeneous reaction of BaP and ozone on soot are 0.015 s^−1^ and 2.8 × 10^−13^ cm^3^, respectively.

The ozone concentration in Shinjuku increased gradually over the course of the study period (Fig. 2), and the average ozone concentration in 2016 was approximately 6 ppb (OP) higher than that in 2007. Because the average ozone concentrations observed in this study period were low (13–25 ppb), it becomes linear in Eq. (4). At low ozone concentrations, the pseudo-first-order rate constant varies considerably, and this variation leads to large differences in PAH degradation rates. Under these conditions, the atmospheric lifetimes of BaP during OP (Fig. 2) were calculated to be 13 min (OP) in 2007 ($[{\text{O}}_{3} ]$ = 13 ppb (OP)) and 9 min (OP) in 2016 ($[{\text{O}}_{3} ]$ = 19 ppb (OP)) (Eq. (4)); that is, the lifetime was shorter in 2016 than that in 2007.

Mu et al.^24^ reported that ${\text{k}}^{\prime}_{1}$ increases almost linearly with increasing temperature. In Tokyo, the annual mean temperature has risen in the 10 years since 2007^25^ owing to the heat island phenomenon. Hence, theoretically, PAHs could be expected to degrade faster in 2016 than in 2007. However, in fact, the concentrations of BaP were almost constant throughout the study period regardless of high or low ozone concentrations (Fig. 1, Fig. S11).

We also evaluated the long-term trend of BaP concentrations during whole period in spring and winter (Fig. S11). Normally, ozone concentrations in urban atmospheres are highest in spring^26^. During our study period, the ozone concentration in the spring (35 ppb) exceeded the annual average (24 ppb) and was low (18 ppb) in winter. However, we observed no differences between the spring and winter trends of PAH concentrations (Fig. S11).

**Figure 4:**
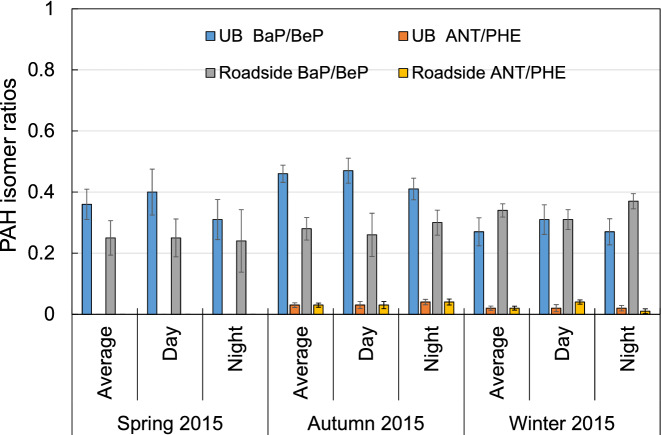
PAH isomer ratios at the urban and roadside sites in 2015.

Here, we discuss the photochemical oxidation reaction using ANT/PHE and BaP/BeP ratio. ANT and BaP are expected to be degraded more easily than their isomers during transport^27^. Thus, high ANT/PHE and BaP/BeP ratios indicate relatively photochemical aging processing. Although BaP/BeP is normally 1 in urban atmospheres, we found that in Shinjuku, BaP/BeP deviated from 1 at the two sites in three seasons (Fig. 4). Low ratios indicate aged PAHs^28,29^. Usually, these ratios are higher in winter and lower in summer. However, at these two sites, these ratios were low and remained constant regardless of the season. Although the decomposition of BaP is promoted by the photochemical oxidation reaction, it is considered that a certain threshold value exists as the degree of the decomposition. According to Brien et al.^30^, which investigated the photolytic aging of secondary organic aerosol (SOA) in a laboratory experiment, there is a certain threshold for its decomposition. Shimada et al.^31^ point out that the coating of SOA is responsible for the existence of the BaP/BeP ratio threshold when transported long distance from China to Okinawa, Japan. They suggests that degradation of PAHs may have been suppressed by SOA coatings. Here, we should investigate the long-term trends of decomposition, but unfortunately BeP measures only 2013 and 2014. At least in central Tokyo, it is considered that photochemical oxidation reaction is promoted more than in ordinary cities because of high ozone concentration. Thus, although the PAH degradation in urban air in Shinjuku could theoretically have increased with the increase in ozone concentration, the presence of an SOA coating may have shielded the PAHs from ozone. Therefore, we suggest that the long-term trend of the PAH concentrations was unlikely to have been affected by PAH degradation by reaction with ozone.

## Implications

We observed PAH concentrations in SPM for 10 years (from 2007 to 2016) in Shinjuku, in central Tokyo. Investigating long-term trends of PAH concentrations in Tokyo, where governmental measures against air pollutants have been implemented, can be expected to improve our understanding of the health effects of air pollution. In this report, we have described our results on the long-term trend of PAH concentrations and the sources of the PAHs. PAH concentrations in urban air in Shinjuku did not change during the study period and did not coincide with the trends of the concentrations of other air pollutants. We suggest two possible explanations for these results: (1) Vehicles were the main sources of the PAHs (accounting for 40 to 80%), and there was no significant change in the contribution of vehicle emissions to PAH concentrations during the period from 2007 to 2016. (2) Although the decomposition of BaP is promoted by the photochemical oxidation reaction, this result suggests that a certain threshold value exists as the degree of the decomposition. In addition to physical loss processes such wet and dry deposition, chemical degradation of PAH in this study can improve chemical loss processes in air quality model.

## Methods

### Air sample collection

Samples for our study were collected at two sites in Shinjuku, which has a population of 330,000. One site was an urban site located on the roof (height, 51 m) of a building on the Nishi-Waseda campus at Waseda University; samples were collected at this site from 2007 to 2016. The other site was a roadside site near the campus, specifically Meiji Street, which is a main prefectural road with high volumes of both diesel- and gasoline-engine vehicles (approximately 40,800 vehicles per day according to a 2015 traffic census^15^). Samples were collected at the roadside site in 2015.

At both sites, SPM was collected on quartz fiber filters (8 × 10 in, 2500QAT-UP, Pallflex) by means of high-volume air samplers (HV-1000F and HV-500F; Sibata, Japan) equipped with an impactor (HV-RW/-1000R/-1000F, Sibata, Japan) stage to eliminate particles with aerodynamic diameters more than 10 µm. The filters were changed every 12 h (Daytime: 6:00 to 18:00, Nighttime: 18:00 to 6:00), and the flow rate was 1 m^3^ min^−1^. To remove organic matter prior to use, we prefired the filters at 600 °C in a muffle furnace for at least 6 h and then conditioned them in desiccators. After sampling, the filters were stored in a freezer (− 20 °C) until analysis.

### Analysis of PAHs

PAHs were extracted from the quartz fiber filters as follows. Each filter was inserted into a 50 mL brown sampling vial and extracted with 25 mL of dichloromethane by ultrasonication (SineSonic2000 SINE, Tokyo, Japan) for 15 min and then the solvent was decanted. This process was repeated three times. The dichloromethane extracts were combined in a 300 mL flask and concentrated to a volume of approximately 3 mL at 213–533 hPa and 305 K in a rotary evaporator (RE400, Yamoto, Tokyo, Japan). Insoluble particles were filtered from the concentrated extract with a disposable syringe filter unit (DISMIS-25HP, Advantec, Tokyo, Japan). Each filtrate was then placed in a 20 mL amber glass vial and evaporated to near dryness under a gentle stream of dry nitrogen gas. As internal standards for evaluating PAH signal intensities, a 100 µL isooctane solution containing a mixture of acenaphthene-*d*_10_ and chrysene-*d*_12_ (5 ppm each) was added to each vial containing a concentrated sample.

PAHs were analyzed by high-performance liquid chromatography on a system consisting of a pump (LC-10AD, Shimadzu, Kyoto, Japan), a fluorescence detector (RF-10AXL, Shimadzu, Kyoto, Japan), a system controller (SCL-10A, Shimadzu, Kyoto, Japan), a degasser (DGU-20A_5_, Shimadzu, Kyoto, Japan), an autosampler injector (SIL-10AD, Shimadzu, Kyoto, Japan), a column oven (CTO-10A, Shimadzu, Kyoto, Japan), and an analytical column (ZORBAX Eclipse PAH (3.0φ × 250 mm) Agilent Technology, State of California, USA). The mobile phase was a 4:1 (v/v) mixture of acetonitrile/water, and the flow rate was 0.5 mL min^−1^. The following 15 PAHs, which had 3–6 rings, were analyzed by means of the selected-ion monitoring method: acenaphthene (ACE), fluorene (FLU), phenanthrene (PHE), anthracene (ANT), fluoranthene (FLT), pyrene (PYR), benz[*a*]anthracene (BaA), chrysene (CHR), benzo[*b*]fluoranthene (BbF), benzo[*k*]fluoranthene (BkF), benzo[*e*]pyrene (BeP), BaP, indeno[1,2,3-*cd*]pyrene (IcdP), DahA, and benzo[*ghi*]perylene (BghiP). Up until 2011, we measured the concentrations of seven PAHs (ANT, FLT, PYR, BkF, BaP, IcdP, and BghiP) and the fifteen PAHs were analyzed after 2012 (ACE, PHE, PYR, BaA, BaP, BbF, BghiP (Sigma Aldrich, St. Louis, USA), ANT, BkF, IcdP (Wako Pure Chemical Industries, Tokyo, Japan), FLT, DahA (Tokyo Kasei Kogyo, Tokyo, Japan), FLU (Kanto Kagaku, Tokyo, Japan), BeP (Accu Standard, Connecticut, USA)). All chemicals were used without further purification. The method detection limits ranged from 77.3 to 347 ng. A clean quartz blank filter was spiked with known amounts of the 15 PAHs to determine recovery yields, which ranged from 79.2 to 98.7%. Trace element analysis and air pollutant data was described in in the Supplement.

### Positive matrix factorization analysis

The positive matrix factorization (PMF) model is a multivariate factor analysis tool that decomposes a matrix of speciated sample data into two matrices: factor contributions and factor profiles. To identify the source types that may contribute to the sample, the user must interpret the factor profiles by using measured source profile information and emission or discharge inventories^32,33^. In this study, the US EPA PMF model (ver. 5.0) was used, and three types of PMF analyses were performed. In the first analysis, we used the concentrations of seven PAHs (7 PAHs: ANT, FLT, PYR, BkF, BaP, IcdP, and BghiP) and 436 samples as input data. In the second, we used the concentrations of fifteen PAHs (15 PAHs: ACE, FLU, PHE, ANT, FLT, PYR, BaA, CHR, BbF, BkF, BeP, BaP, IcdP, BghiP, and DahA) and 254 samples as input data. In the third, we used data for 21 species (Al, V, Cr, Mn, Fe, Ni, Cu, Zn, Cd, Pb, As, FLT, PYR, BaA, CHR, BbF, BkF, BaP, IcdP, BghiP, and DahA) and 254 samples as input data. We describe the source profile information in the Supplement (Figs. S1–S3).

We selected the factor solution that had the most interpretable results, on the basis of Sugiyama et al.^34^; Miura et al.^15^ (Tables S1–S6). The criteria for choosing a factor number were the interpretability of the factors and the results of error estimation. We describe the procedure for error estimation in the Supplement.

The results of PMF analysis have uncertainty because the magnitude of the contribution of a given major source may change during the study period. Thus, the PMF model was applied to two datasets. First, the measurement results for all the observation periods in a given year were used as a single dataset to estimate the major factors. Second, the measurement results for the observation periods in a given year were modeled separately (see Figs. S4–S10 in the Supplement).

## Supplementary Information

Supplementary Information.
