# Supplementary material for: Effects of changes in polycyclic aromatic hydrocarbons (PAHs) emissions and degradation on their concentrations in Tokyo from 2007 and 2016
Source: Sci Rep. 2022 Mar 11;12:4249. doi: 10.1038/s41598-022-08138-8 (PMC8917142; doi:10.1038/s41598-022-08138-8)
Supplement: Supplementary file 1 — Supplementary Information. [file 41598_2022_8138_MOESM1_ESM.docx]

**Effects of Changes in Polycyclic Aromatic Hydrocarbons (PAHs) Emissions and Degradation on Their Concentrations in Tokyo from 2007 and 2016**

Kojiro Shimada^1,4^, Masayuki Nohchi^1^, Koji Maeshima^1^, Tomonori Uchino^1^, Yusuke Kobayashi^1^, Kazuki Ono^1^, Hiroko Ogata^1^, Naoya Katsumi^1,2^, Koji Inazu^3^, Hiroshi Okochi^1^

^1^School of Creative Science and Engineering, Waseda University, 3-4-1 Okubo, Shinjuku, Tokyo, 169-8555 Japan

^2^Department of Bioresources and Environmental Sciences, Ishikawa Prefectural University, 1-308 Suematsu, Nonoichi, Ishikawa 921-8836, Japan

^3^National Institute of Technology, Numazu College, 3600 Ooka, Numazu City, Shizuoka, 410-8501 Japan

^4^Present address: Department of Chemistry, Biology, and Marine Science, University of the Ryukyus, Okinawa 903-0213 Japan

Corresponding author: Hiroshi Okochi (hokochi@waseda.jp)

Address: School of Creative Science and Engineering, Waseda University, 3-4-1 Okubo, Shinjuku, Tokyo, Japan

**◼ EXPERIMENTAL METHODS Trace element analysis and air pollutant data**

The methods for trace element analysis have been described previously.^1^ SPM for trace element analysis was collected on quartz fiber filters with a high-volume air sampler (Sibata, HV-1000F, Tokyo, Japan) at a flow rate of 1 m^3^ min^−1^. The filters were changed every 12 h. Trace metals were measured by means of inductively coupled plasma mass spectrometry (Agilent 7500 and Agilent 7700). We here defined eight trace elements such as Pd, Cd, Zn, Cu, Ni, Mn, Cr, and V among them as anthropogenic trace elements because their enrichment factors, which were calculated using aluminum as an indicator of earth crust, were larger than 100.^2^

We did not measure the concentration of air pollution such as NO*_x_*, ozone, and PM_2.5_ at our sampling site during the study periods, so we used the measured values of them at the closest ambient air monitoring stations. For NO*_x_*, and ozone concentrations, we used data collected 33 m above sea level at the Shinjuku Office Measurement Bureau (1-4-1 Kabukicho Shinjuku, Tokyo), which is the air monitoring station closest to our study site.^3^

PM_2.5_ concentrations, we used data collected 20 m above sea level at the Shinjuku Office Measurement Bureau (2-2 Tsukasamachi Kanda Chiyodaku, Tokyo), which is the air monitoring station closest to our study site.^4^ We used the following two datasets for NO*_x_*, and ozone: We used all data set of NO*_x_* and ozone in the whole period and selected data from all data sets in the same measurement period described in Table 1.

**Positive Matrix Factorization Analysis.** The Positive Matrix Factorization (PMF) model is a multivariate factor analysis tool that decomposes a matrix of speciated sample data into two matrices: factor contributions and factor profiles. The factor profiles need to be interpreted by the user to identify the source types that may contribute to the sample by using measured source profile information and emission or discharge inventories.^5^

The mathematical model in its matrix is:

$E=X-G*F$ (S1)

Where *X* is the measurement concentrations dataset matrix, *G* is the source contribution matrix, *F* is the source profile matrix, and *E* is the residual matrix.

To determine the optimal number of factors, it is necessary first to determine the minimum *Q* values for different numbers of factors. *Q* function defined as:

$Q\left( E \right)=\sum_{i=1}^{m} \sum_{j=1}^{n} \left( E_{\mathrm{ij}}/\sigma_{\mathrm{ij}} \right)^{2}$ (S2)

Where *X* is the chemical component dataset matrix, *G* is the source contribution matrix, *F* is the source profile matrix, and *E* is the residual matrix.

The values $\sigma_{ij}$ are the standard deviation of the measurement concentrations value *X.* The task of the non-negatively constrained weighted factor analysis is: Minimize *Q(E)* with respect to *G* and *F* under the constraint that all or some of the elements of *G* and *F* are constrained to non-negative values. More details on PMF were described in Norris et al.^6^

Different *Q* functions can be defined that *Q* (true) is the goodness of fit parameter calculated including all points. *Q* (robust) is the goodness of fit parameter calculated excluding points not fit by the model. The reduction in *Q* with the increase in the number of factors and the agreement of estimated *Q* with its theoretical value, *Q* (theory), were used to identify possible optimal solutions.

　$Q_{\mathrm{theory}}=nm-p\left( n+m \right)$ (S3)

Where *n* is number of samples, *m* is number of chemical components and *p* is number of factors.^7^

In this study, to identify the source categories through PMF analysis, we first determined 1) the optimal number of factors and 2) the stability and uncertainty of the solution. To do that, we evaluated the results of 1) base runs and 2) a bootstrap run and displacement approach (DISP). The error fraction was set to 0.15. An additional uncertainty of 15 % was added to each value. For missing values, the median values of these components were used, and their errors were estimated at four times the species-specific median. If the concentration was less than or equal to the detection limit, the uncertainty was calculated as 5–6 times the detection limit.^6^ Subsequently, the signal-to-noise (S/N) ratio for all data was examined to ascertain whether the measurement variability was real or within the noise level^6^; species with S/N ratios greater than 1 were considered to be “strong.” All species were set as “strong.”

To determine the optimal number of factors, various numbers of factors were tested and the resultant PMF calculation results were evaluated.^8^ We performed 20 random runs and retained the runs that produced minimum *Q* values for 3–10 factors in base runs.

**15 PAHs dataset.** A five-factor solution was selected on the basis of the most physically interpretable results with the least factor smearing and the residuals that were the most normal. To estimate the stability and uncertainty in factor contributions, we performed a bootstrap model analysis and DISP. Tables S1 and S2 summarize the bootstrap analysis and DISP, respectively. Bootstrap analysis was performed 100 times with a minimum correlation value (*R*) of 0.6. The average percentage of bootstrap factors mapped back to the original PMF factors of this study was 90 % (range 83–97 %; Table S2), which was higher than that reported by Callén et al. (average 79%, range 74–90 %).^9^ This result indicates that our factor profile solutions were sufficiently unique. No swaps were present in any of the factors for d*Q*max 4 and 8 (Table S1). Thus, the solution was stable. These error estimations demonstrate that the model simulation results were acceptable.

Table S1. Swaps according to factors in DISP (15PAHs dataset).

|  | | | | | |
| --- | --- | --- | --- | --- | --- |
|  | Factor 1 | Factor 2 | Factor 3 | Factor 4 | Factor 5 |
| dQmax = 4 | 0 | 0 | 0 | 0 | 0 |
| dQmax = 8 | 0 | 0 | 0 | 0 | 0 |

Table S2. Percentage of bootstrap factors mapped back to the original PMF factors from the five-factor PMF solution (15PAHs dataset).

|  | | | | | | | |
| --- | --- | --- | --- | --- | --- | --- | --- |
| Boot　Factor | Factor 1 | Factor 2 | Factor 3 | Factor 4 | Factor 5 | Unmapped | % Bootstrap factors  mapped to original PMF factors |
| 1 | 85 | 1 | 1 | 11 | 2 | 0 | 85% |
| 2 | 1 | 92 | 1 | 4 | 2 | 0 | 92% |
| 3 | 0 | 0 | 97 | 1 | 2 | 0 | 97% |
| 4 | 3 | 2 | 1 | 83 | 11 | 0 | 83% |
| 5 | 1 | 1 | 1 | 5 | 92 | 0 | 92% |

**PAHs+elements.** Unfortunately, when we used a dataset for 15 PAHs and 11 trace elements, uncertainty of bootstrap factors was high. In this study, we used a dataset for 10 PAHs and 11 trace elements. A six-factor solution was selected on the basis of the most physically interpretable results with the least factor smearing and the residuals that were the most normal. To estimate the stability and uncertainty in factor contributions, we performed a bootstrap model analysis and DISP. Tables S3 and S4 summarize the bootstrap analysis and DISP, respectively. Bootstrap analysis was performed 100 times with a minimum *R* value of 0.6. The average percentage of bootstrap factors mapped back to the original PMF factors of this study was 74 % (range 64–77 %; Table S4). This result indicates that our factor profile solutions were sufficiently unique. No swaps were present in any of the factors for d*Q*max 4 and 8 (Table S3). Thus, the solution was stable. These error estimations demonstrate that the model simulation results were acceptable.

Table S3. Swaps according to factors in DISP (PAHs+elements).

|  | | | | | |  |
| --- | --- | --- | --- | --- | --- | --- |
|  | Factor 1 | Factor 2 | Factor 3 | Factor 4 | Factor 5 | Factor 6 |
| dQmax = 4 | 0 | 0 | 0 | 0 | 0 | 0 |
| dQmax = 8 | 0 | 0 | 0 | 0 | 0 | 0 |

Table S4. Percentage of bootstrap factors mapped back to the original PMF factors from the six-factor PMF solution (PAHs+elements).

|  | | | | | | | | |
| --- | --- | --- | --- | --- | --- | --- | --- | --- |
| Boot Factor | Factor 1 | Factor 2 | Factor 3 | Factor 4 | Factor 5 | Factor 6 | Unmapped | % Bootstrap factors mapped to original PMF factors |
| 1 | 77 | 0 | 0 | 0 | 0 | 0 | 23 | 77% |
| 2 | 1 | 64 | 1 | 1 | 6 | 3 | 24 | 64% |
| 3 | 0 | 1 | 73 | 1 | 2 | 0 | 23 | 73% |
| 4 | 0 | 0 | 0 | 77 | 0 | 0 | 23 | 77% |
| 5 | 0 | 0 | 1 | 0 | 76 | 0 | 23 | 76% |
| 6 | 1 | 0 | 0 | 0 | 2 | 74 | 23 | 74% |

**7 PAHs dataset.** A four-factor solution was selected on the basis of the most physically interpretable results with the least factor smearing and the residuals that were the most normal. To estimate the stability and uncertainty in factor contributions, we performed a bootstrap model analysis and DISP. Tables S5 and S6 summarize the bootstrap analysis and DISP, respectively. Bootstrap analysis was performed 100 times with a minimum *R* value of 0.6. The average percentage of bootstrap factors mapped back to the original PMF factors of this study was 96 % (range 86–99 %; Table S4). This indicates that our factor profile solutions were sufficiently unique. No swaps were present in any of the factors for d*Q*max 4 and 8 (Table S5). Thus, the solution was stable. These error estimations demonstrate that the model simulation results were acceptable.

Table S5. Swaps according to factors in DISP (7 PAHs dataset).

|  | | | | |
| --- | --- | --- | --- | --- |
|  | Factor 1 | Factor 2 | Factor 3 | Factor 4 |
| dQmax = 4 | 0 | 0 | 0 | 0 |
| dQmax = 8 | 0 | 0 | 0 | 0 |

Table S6. Percentage of bootstrap factors mapped back to the original PMF factors from the four-factor PMF solution (7 PAHs dataset).

|  | | | | | | |
| --- | --- | --- | --- | --- | --- | --- |
| Boot Factor | Factor 1 | Factor 2 | Factor 3 | Factor 4 | Unmapped | % Bootstrap factors mapped to original PMF factors |
| 1 | 99 | 0 | 1 | 0 | 0 | 99% |
| 2 | 0 | 99 | 1 | 0 | 0 | 99% |
| 3 | 0 | 1 | 99 | 0 | 0 | 99% |
| 4 | 1 | 1 | 1 | 96 | 0 | 86% |

**PAH concentration profiles.** Seasonal variations in the Σ7PAH and Σ15PAH concentrations are shown in Fig. S1. The overall average Σ7PAH concentrations in spring (*N* = 65), summer (*N* = 109), autumn (*N* = 41), and winter (*N* = 39) were 0.92, 0.95, 1.06, and 1.54 ng m^–3^, respectively; and the overall average Σ15PAH concentrations in spring (*N* = 65), summer (*N* = 109), autumn (*N* = 41), and winter (*N* = 39) were 1.60, 1.91, 1.96 and 2.91 ng m^–3^, respectively. The Σ7PAH concentrations accounted for 58 % (spring), 50 % (summer), 54 % (autumn), 53 % (winter) of the Σ15PAH concentrations, respectively, that is, >50% in all four seasons. For both sets of PAHs, the average concentrations were highest in winter, but no other clear seasonal variation was observed for either set. It has been reported that PAH concentrations at urban sites are generally high in winter and low in summer.^10^

PAH concentrations are affected by dispersion, deposition, photochemical degradation, and heterogeneous reactions with ozone, whereas the concentrations of anthropogenic elements are affected only by dispersion and deposition. Therefore, the elements are more appropriate tracers than are PAHs because the concentrations of elements are not decreased by chemical reactions during transport.

Figure S2 shows the seasonal variation of anthropogenic trace elements and vehicle-derived trace elements such as Zn and Cu. Zinc is an indicator of motor oil additives^11^ while Cu is attributed to brake pad abrasion.^12^ Among the rest of anthropogenic trace elements, V and Ni are known to be emitted during the combustion of marine fuel oil^13^ while Cd and Pb are possible indicators of waste incineration at our sampling site.^14^ As shown in Fig. S2, Zn was the most abundant anthropogenic trace elements in all seasons and contributed to 66 % in spring, 67 % in summer, 67 % in autumn, and 75 % in winter, respectively. Both the concentrations of anthropogenic trace elements and vehicle-derived trace elements were the highest in winter. This result can be attributed to the fact that vehicle emissions are affected by ambient temperature; in particular, emission of particulate matter from gasoline vehicles is high during cold starts.^15^ Thus, our results indicate that vehicle emissions were a major source of anthropogenic trace elements in the urban atmosphere at Shinjuku.

We investigated PAH concentration profiles to clarify the source of the PAHs in the collected aerosols. The PAHs analyzed in this study can be classified according to the number of aromatic rings: three-ring PAHs (ACE, FLU, PHE, ANT), four-ring PAHs (FLT, PYR, BaA, CHR), five-ring PAHs (BbF, BkF, BeP, BaP, DahA), and six-ring PAHs (IcdP, BghiP). Liu et al.^16^ classified three-rings PAHs into low molecular weight (LMW), four-rings PAHs into medium molecular weight (MMW), and five- and six-rings PAHs high molecular weight (HMW). In this study, the HMW PAHs accounted for 60, 59, 59, and 59 % of the average Σ7PAH concentrations in spring, summer, autumn, and winter, respectively (Fig. S3). This result is consistent with those of Liu et al.,^16^ who reported that HMW PAHs account for as much as 64–70 % of the total PAH concentration in Guangzhou City, China, which is known for its heavy traffic.


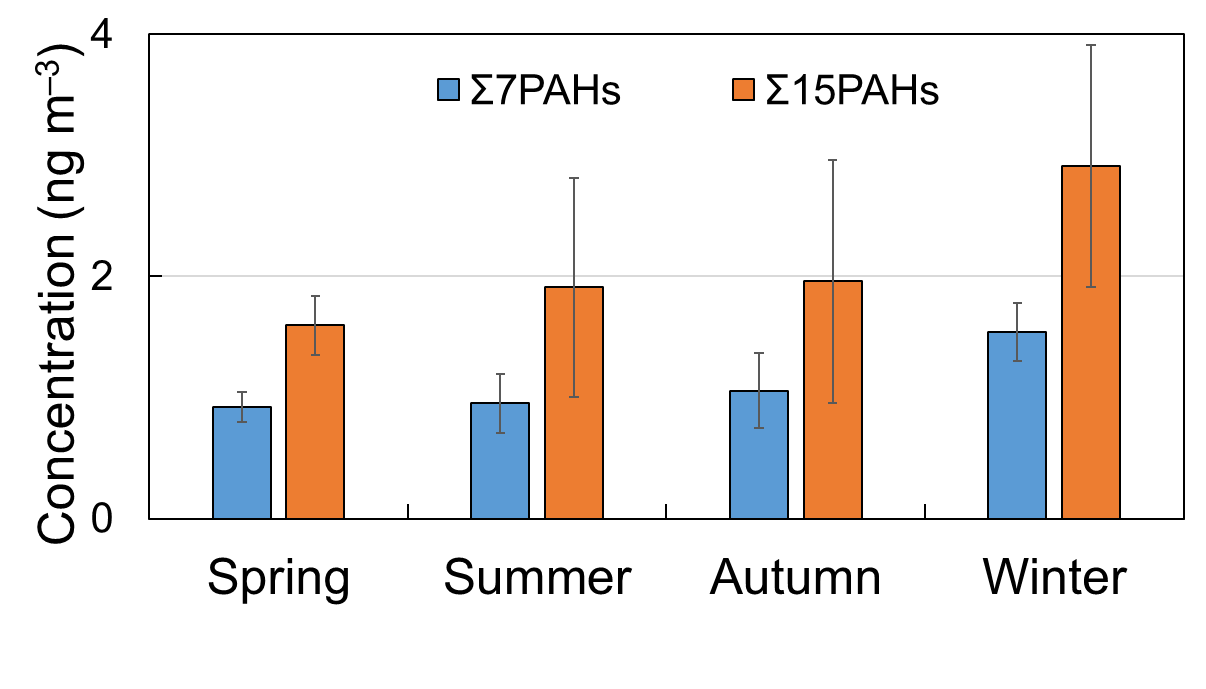


Figure S1. Seasonal variations of average ΣPAH concentrations, 2007–2016.


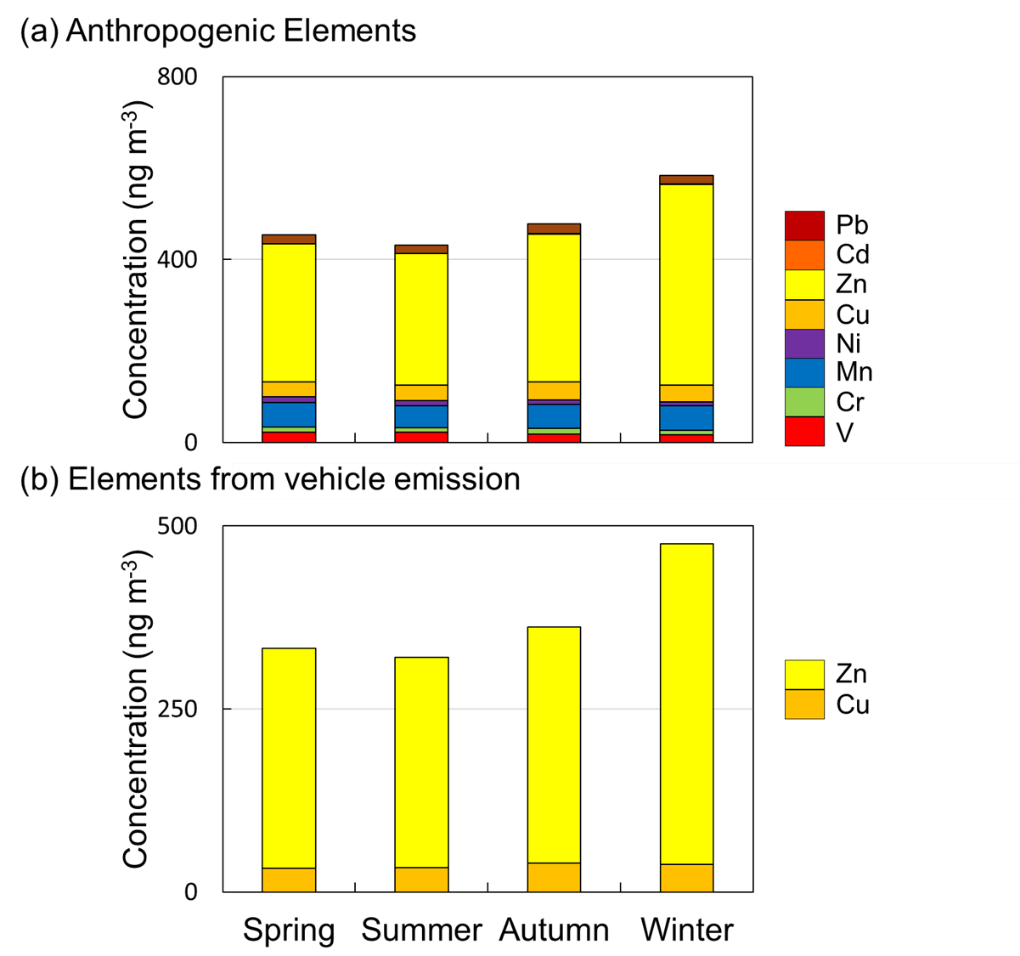


Figure S2. Seasonal variations of (a) anthropogenic element concentrations and (b) concentrations of elements present in vehicle emissions, 2011–2016.


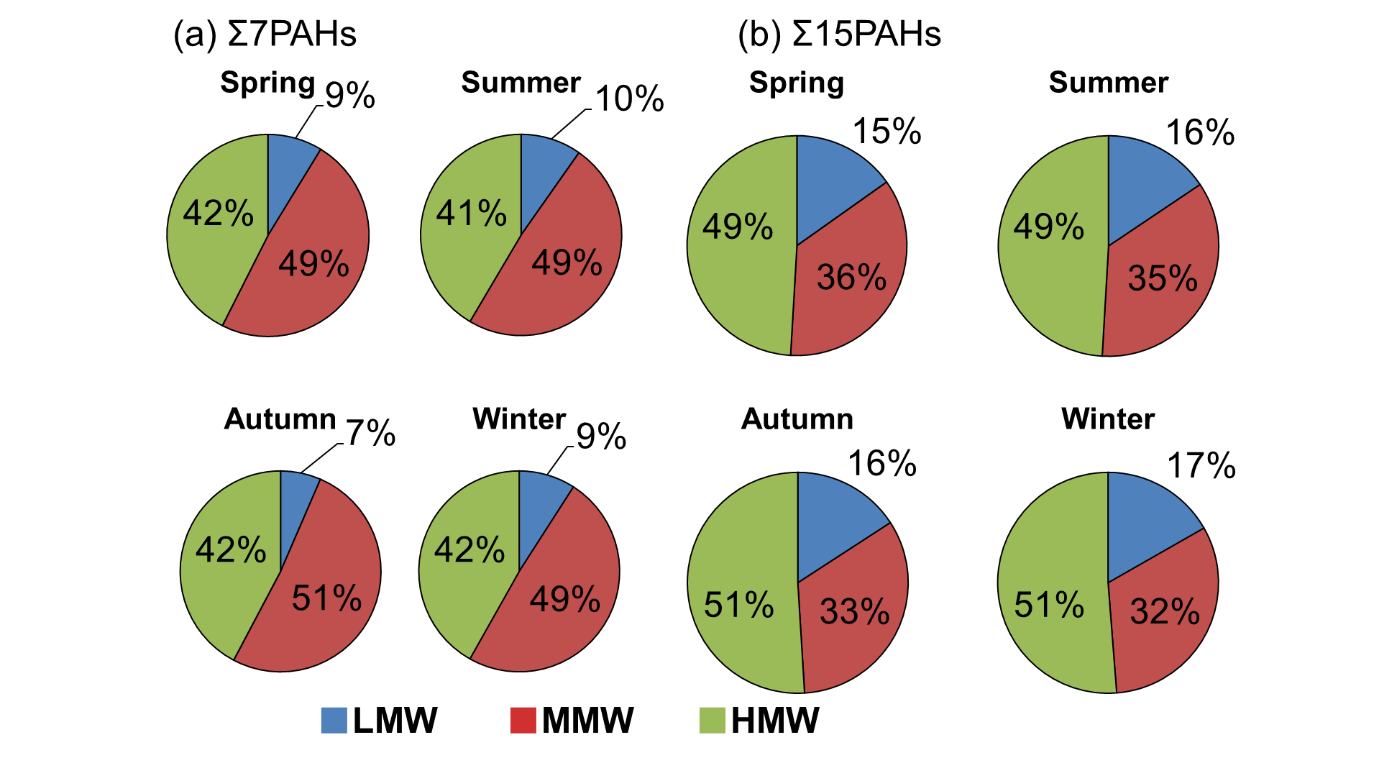


Figure S3. Seasonal variation of proportions of LMW, MMW, and HMW PAHs: (a) Σ7PAHs and (b) Σ15PAHs.

**Factor identification** **(15PAHs)**. We carried out a PMF analysis with 15 PAHs dataset in SPM collected from 2012 to 2016. This analysis identified five factors (Fig. S4). Factor 1 was dominated by the HMW PAHs IcdP, BghiP, and DahA, which are typical markers for gasoline vehicle emissions.^17,18^ The PAH profile for factor 1 was similar to that for gasoline vehicle emissions.^18－20^ Therefore, we assigned factor 1 to these emissions. Factor 2 was dominated by PYR, CHR, BbF, BkF, and BaP. PYR is known to be a marker for diesel vehicle emissions.^21^ In addition, the profile for factor 2 exhibited more three- to four-ring PAH characteristics than did the profile for factor 1. Diesel vehicle emissions are reported to be richer in PYR and BkF than are gasoline vehicle emissions.^22, 23^ Therefore, we assigned factor 2 to diesel vehicle emissions. Factor 3 was dominated by BeP, BbF, BkF, BaP and IcdP, and the profile for factor 3 was similar to that reported for waste incineration.^24,25^ Therefore, we assigned factor 3 to waste incineration. Factor 4 was dominated by ACE, FLU, PHE, ANT, and FLT. Heavy oil combustion has been reported to be associated with high concentrations of relatively volatile PAHs, such as LMW PAHs.^26, 27^ We assigned factor 4 to heavy oil combustion. Factor 5 was dominated by ANT, FLT, PYR, BaA, and CHR. FLT, PYR, and CHR are known to be markers of coal combustion,^19, 20^ but FLT and PYR are also emitted during biomass burning.^28^ The sources of coal combustion in Tokyo are coal boilers,^29^ and the sources of biomass burning are wood boilers and smoking.^29^ However, because the contribution of factor 5 was low (8 %), we suggest that it can be attributed to a combination of other minor sources, such as coal combustion and biomass burning, and we designated factor 5 as “Others.”


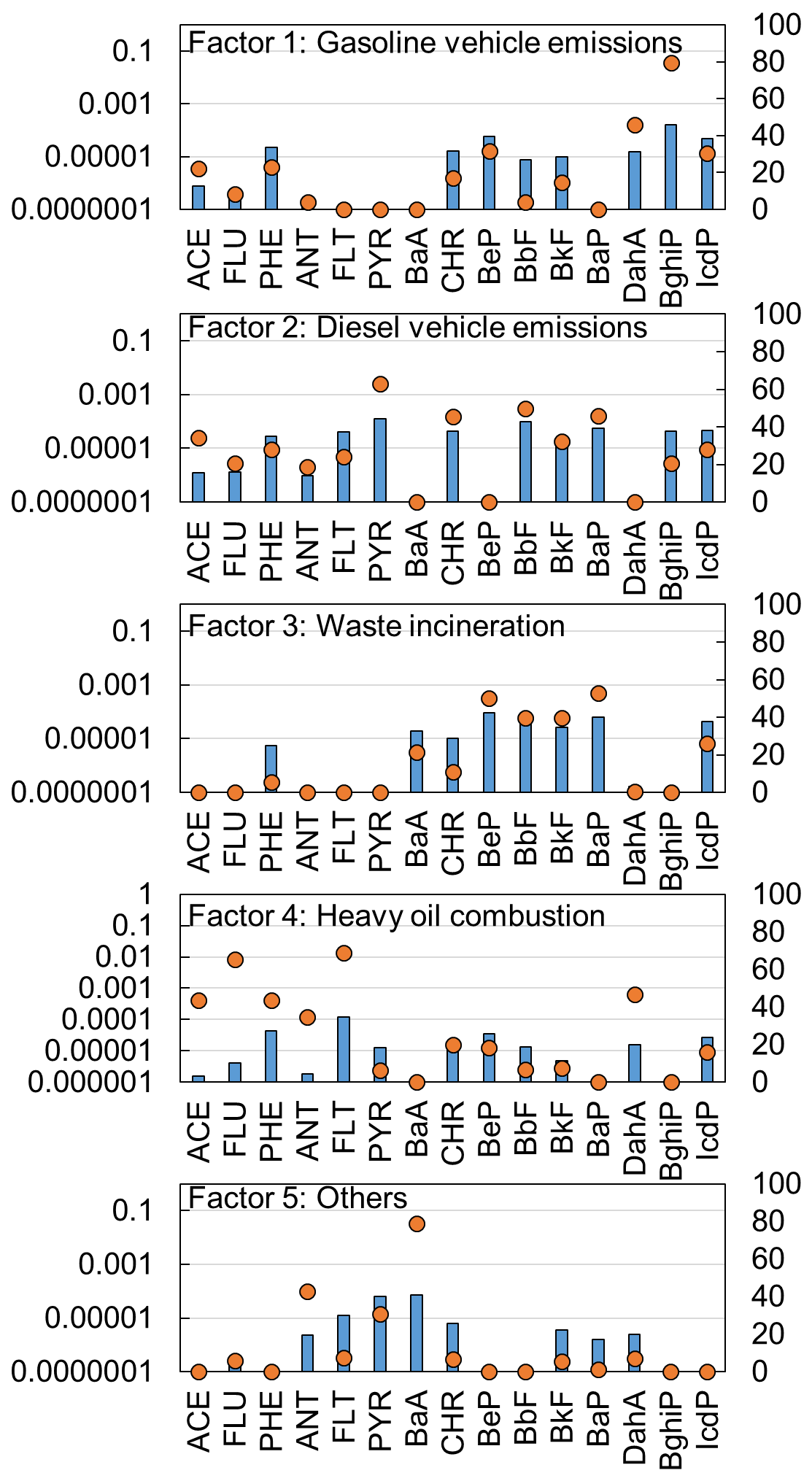


Concentration of Species

% of Species

Figure S4. Source profiles for the five factors calculated by PMF. The bars show the concentration of each PAH and the concentration apportioned to the factor  (left axis), and the circles show the percentage contribution of each PAH (right axis).

**Factor identification** **(PAHs+elements).** In the PMF analysis, we used datasets for two components: PAHs and trace elements. The daily concentrations of metals (Al, V, Cr, Mn, Fe, Ni, Cu, Zn, As, Cd, and Pb), which were observed at the same time as the PAH concentrations (FLT, PYR, BaA, CHR, BbF, BkF, BaP, IcdP, BghiP, and DahA), were used in the analysis. Data from 2012–2016 were used for this analysis. Six factors were identified by PMF (Fig. S5).

The profile of factor 1 was dominated by Al and Fe, which are tracers of soil and road dust (Gietl et al., 2010; Harrison et al., 2012).^30, 29^ Thus, factor 1 was assigned to soil and road dust.

Factor 2 was dominated by Cu, Zn, and high-molecular-weight PAHs such as IcdP and DahA. High loadings of Cu, Sn, and Ba have been attributed to brake pad abrasion.^30, 31^ Zn is an indicator of motor oil additives.^11^ High-molecular-weight PAHs such as IcdP and DahA are tracers of gasoline vehicle emissions (Miguel et al., 1998; Zechmeister et al., 2006).^31,32^ Thus, factor 2 was assigned as gasoline and diesel vehicle emissions.

Factor 3 was dominated by Cd, Pb, FLT, BaA, CHR, BkF, BbF, BaP, and BghiP. In Tokyo, Zn, Cd, and Pb are known to be markers for waste incineration.^34,14^ Similar profiles have been reported for waste incineration.^24,25^ Thus, factor 3 was assigned to waste incineration.

Factor 4 was dominated by V and Ni, which are emitted from the combustion of marine fuel oil. Particles emitted from the diesel engines of ships are also known to be composed mainly of organic carbon, sulfate, and ash. Vanadium and Ni are major components of ash and are formed by burning of low-grade fuel oils.^13,35^ Factor 4 showed a seasonal trend: high in summer and low in winter. However, both V and Ni concentrations were the highest in winter (Fig. S2). Thus, factor 4 was assigned to heavy oil combustion.

Factor 5 was dominated by Cr, Mn, Fe, and Ni. Cr, Mn, Fe and Ni are known to be emitted by the iron and steel industry.^29^ Thus, factor 6 was assigned to emissions from the iron and steel industry.

Factor 6 was dominated by As, PYR, CHR, BbF, and BkF, which are markers for coal combustion.^20,36,37^ PYR and CHR are also emitted during biomass burning.^28^ In Tokyo, coal fuel boilers are the source of emissions due to coal burning,^29^ and wood combustion boilers and smoking are the sources of emissions due to biomass burning.^29^ Because the contribution of factor 6 was low (4 %), we attributed it to a combination of other minor sources (such as coal combustion and biomass burning). We designed factor 6 as “Others.”


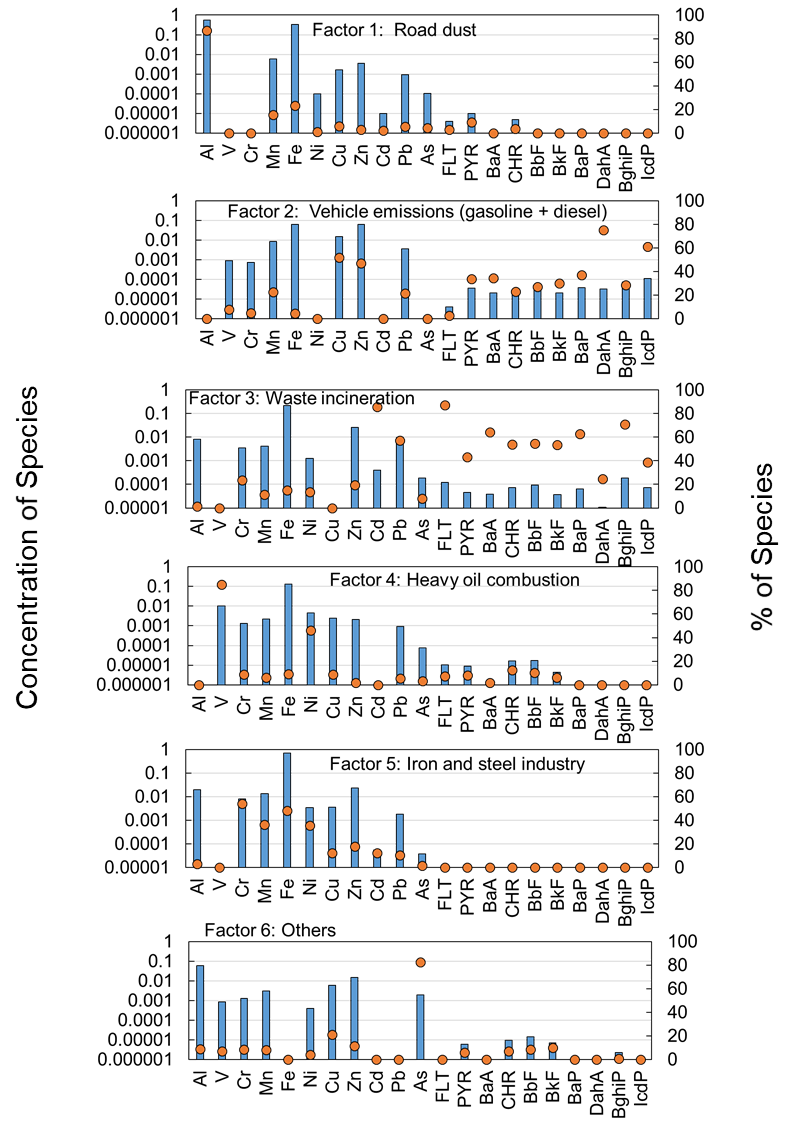


Figure S5. Source profiles for the six factors identified by PMF. The bars show the concentration of each PAH and the concentration apportioned to the factor (left axis), and the circles show the percentage contribution of each PAH (right axis).

**Factor identificatio**n **(7 PAHs dataset).** We used data for 7 species of PAHs (ANT, FLT, PYR, BkF, BaP, IcdP, and BghiP) from 2007 to 2016 in the PMF analysis, which identified four factors (Gasoline vehicle emissions, Diesel vehicle emissions, Heavy oil combustion Waste incineration) according to the method described above (Fig. S6).


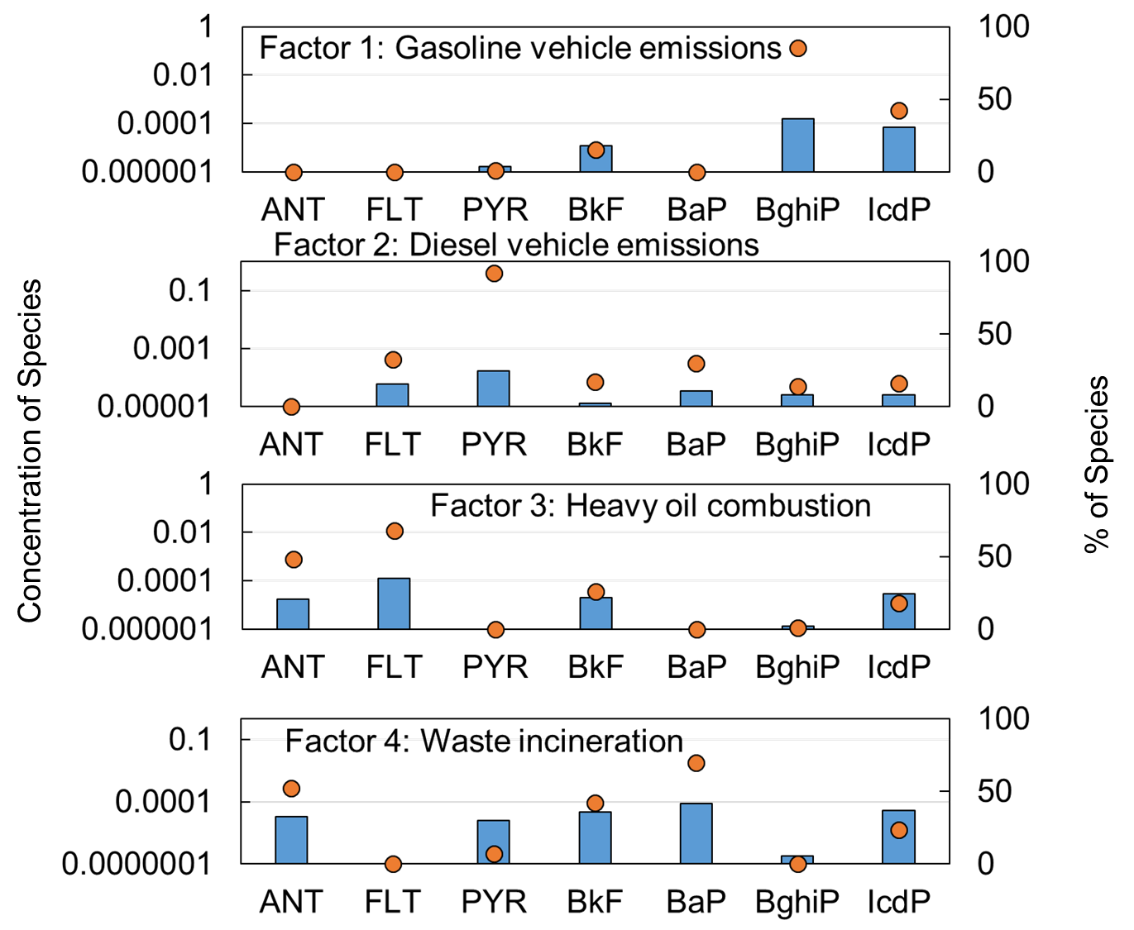


Figure S6. Source profiles for the four factors identified by PMF. The bars show the concentration of each PAH and the concentration apportioned to the factor (left axis), and the circles show the percentage contribution of each PAH (right axis).

**Investigating the common factors in PMF by comparing the results for the dataset of 15 PAHs with the results for PAHs+elements.** We used the following three datasets for PMF analysis: 15PAHs, 7PAHs, and PAHs+elements. To investigate the long-term trend of the contribution ratio to each PAH sources during 10 years from 2007 to 2016, we used the concentrations of 7 PAHs. However, we suspected that identifying PAH sources by means of PMF might be difficult because of the lack of information on sources of each PAHs. In order to use the dataset of 7 PAHs, we investigated the common factors in PMF by comparing the results for the dataset of 15 PAHs with the results for PAHs+elements. Then we verified whether 7 PAHs dataset could be used to investigate the long-term trend of the contribution to each PAH sources by comparing results of 15 PAHs dataset based on the common factors identified using PMF analysis.

First, we carried out a PMF analysis with 15 PAHs dataset in SPM collected from 2012 to 2016. Then, using the procedure described by Han et al.,^38^ we compared the source profiles of the five factors identified with the 15 PAHs dataset (Fig. S7) and the six factors identified with the dataset of PAHs+elements (Fig. S8), either by inputting all the data for a given year as a single file or by inputting the data for each observation period as a separate file, with the goal of understanding the uncertainty of the PMF results.

The source profiles for 15 PAHs dataset and dataset of PAHs+elements were similar, regardless of whether the data were inputted as a single file or as multiple files (Figs. S7 and S8). This similarity indicates that the magnitude of the contribution from a major source did not change from observation period to observation period. Therefore, we used the single-file-input method.

We compared vehicle emissions (both diesel and gasoline), waste incineration, and heavy oil combustion, which were identified as sources shared in common by both datasets. The common source of IcdP and DahA was dominated by gasoline vehicle emissions between Σ15 PAHs and PAHs+elements. The common source of PYR, CHR, BbF, BkF, and BaP was dominated by diesel vehicle emissions between Σ15PAHs and PAHs+elements. The common source of BkF and BbF was dominated by waste incineration between Σ15 PAHs and PAHs+elements. The common source of FLT was dominated by heavy oil combustion between Σ15PAHs and PAHs+elements.

Finally, the dataset of 7 PAHs collected from 2007 to 2016 were used in the PMF analysis, which identified four factors (Fig. S9). Here again, following Han et al., ^38^ we compared the source profiles of the four factors by inputting the entire dataset for a given year and by inputting the data for each observation period separately to assess the uncertainty in the PMF results. The source profiles for the 7 PAHs dataset were similar, regardless of the data input method (Fig. S9), which indicates that the magnitude of the contribution from a major source did not change not depending on any observation period from 2007 to 2016. Therefore, we carried out the analysis by inputting the data as a single file.

We compared gasoline vehicle emissions, diesel vehicle emissions, heavy oil combustion, and waste incineration, which were identified as sources shared in common by the datasets of 15 PAHs and the 7 PAHs. The common source of IcdP was dominated by gasoline vehicle emissions while that of PYR was dominated by diesel vehicle emissions. The common source of ANT and FLT was dominated by heavy oil combustion and that of BkF was dominated by waste incineration. The factor profiles for the 7 PAHs dataset were consistent with the profiles for the 15 PAHs dataset. Hence, we found that the behaviors of both dataset before 2012 were consistent with each other.

To better understand the long-term trend of the PAH concentrations, we evaluated the trends of the contributions of the major sources. Figure 8 shows variations of the contributions of vehicle emissions (gasoline + diesel) for the datasets of 7 PAHs, 15 PAHs, and PAHs+elements. Using the datasets of 7 PAHs and 15 PAHs, vehicle emissions accounted for 40–80 % of the total PAHs. Whereas vehicle emissions contributed 20–50 % using the dataset of PAHs+elements. The contributions of vehicle emissions using the datasets of PAHs+elements and 15 PAHs were similar from 2012 to 2016, except for in 2015. The contribution of vehicle emissions showed no seasonal variation (Fig. S10). Therefore, the PAH concentration profiles and the PMF analysis indicated that the PAHs observed from 2007 to 2016 originated mainly from vehicles and that the long-term trend of the PAH concentrations was affected by changes in the contributions of vehicle emissions.


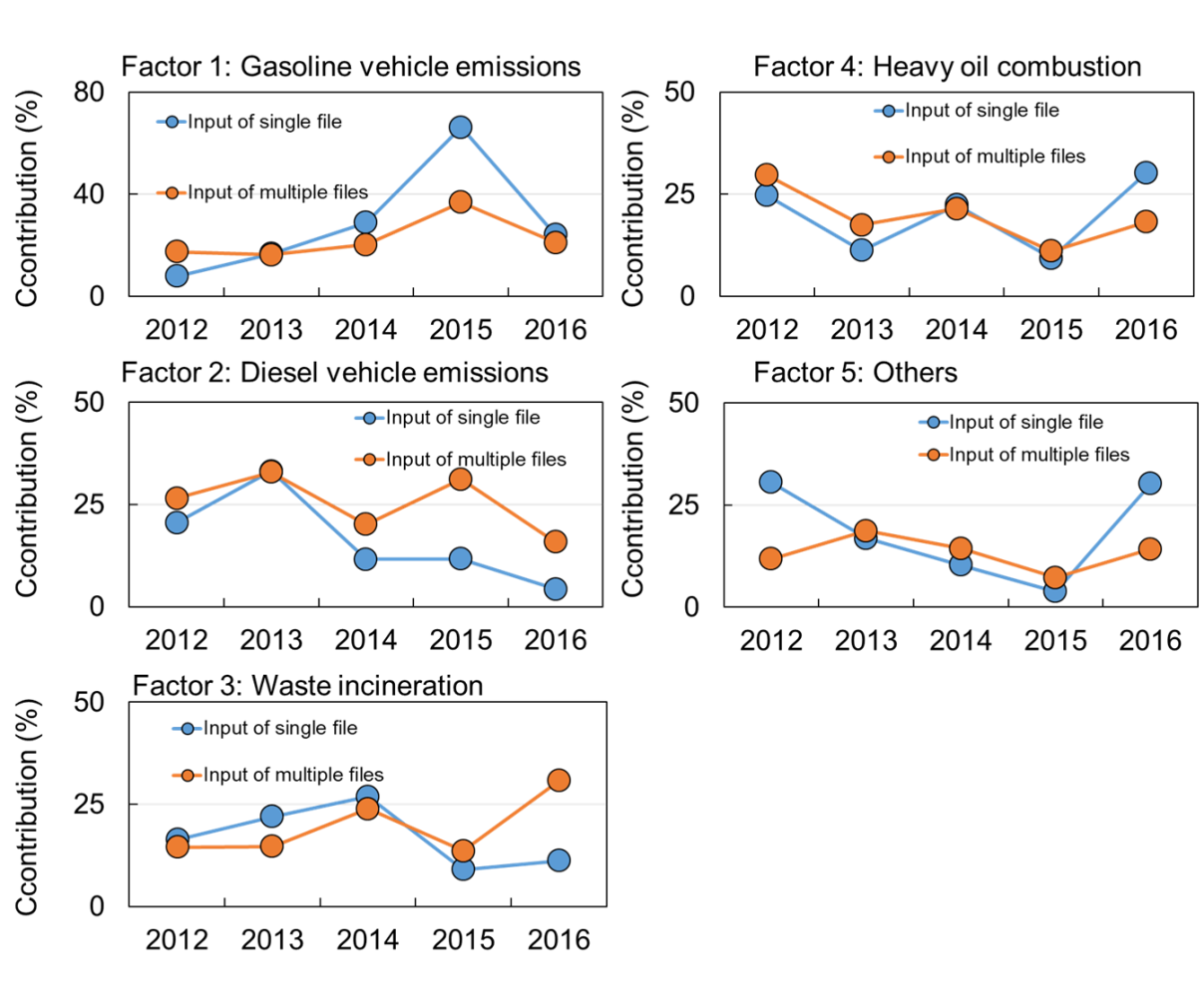


Figure S7. Contributions of each factor as indicated by modeling results obtained by inputting a single file for all observation periods in a given year or by inputting a separate file for each observation period during a given year (15 PAHs dataset).


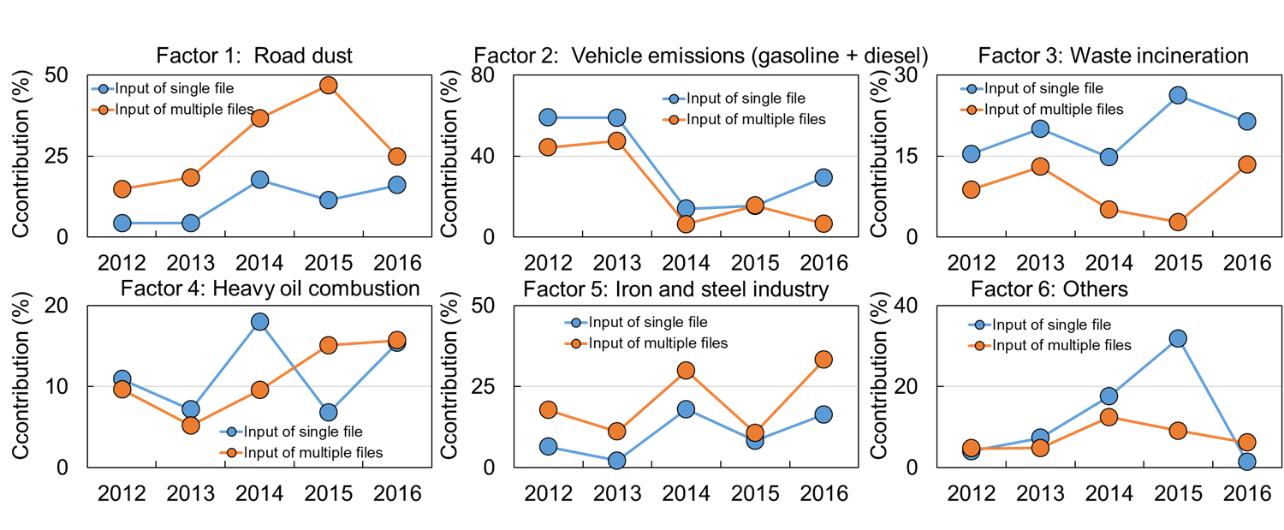


Figure S8. Contributions of each factor as indicated by modeling results obtained by inputting a single file for all observation periods in a given year or by inputting a separate file for each observation period during a given year (PAHs+elements).


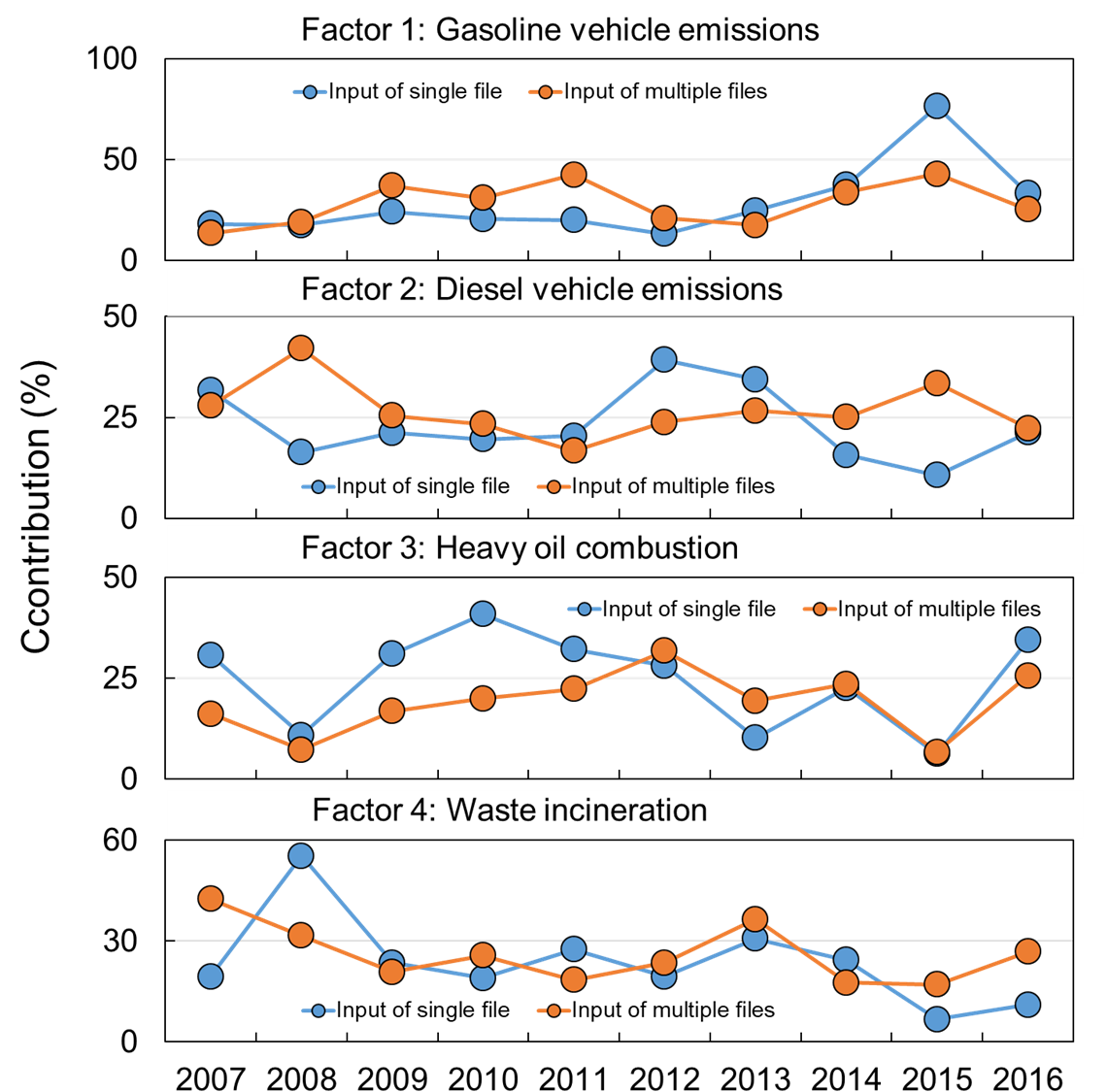


Figure S9. Contributions of each factor as indicated by modeling results obtained by inputting a single file for all observation periods in a given year or by inputting a separate file for each observation period during a given year (7PAHs dataset).


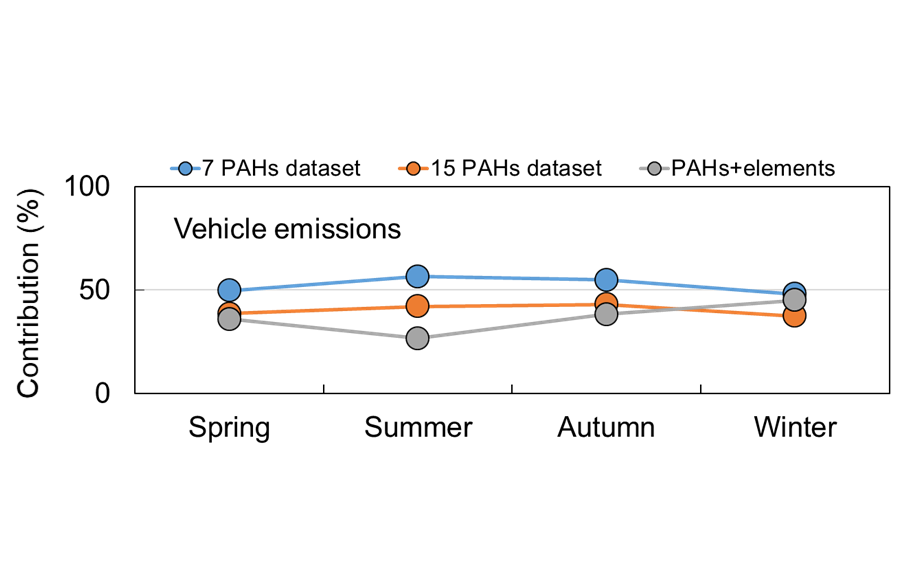


Figure S10. Seasonal variation of the contribution of vehicle emissions, 2012–2016.

Figure S11. Annual Σ7PAH concentrations at the urban site during whole, daytime and nighttime period, 2009-2015es in (a) spring and (b) winter.

**REFERENCES**

1. Shimada, K., Shimada, M., Takami, A., Hasegawa, S., Fushimi, A., Arakaki, T., Arakaki, T., Izumi, W., Hatakeyama, S., 2015. Mode and place of origin of carbonaceous aerosols transported from East Asia to Cape Hedo, Okinawa, Japan, Aerosol Air Qual. Res., 15, 799-813, doi: 10.4209/aaqr.2014.09.0190.
2. Gao, Y., Nelson, D.E., Field, P.M., Ding, Q., Li, H., Sherrell, R.M., Gigliotti, C.L., Ryda, V., Glenn, T.R., Eisenreich, S.J., 2002. Characterization of atmospheric trace elements on PM2.5 particulate matter over the New York–New Jersey harbor estuary, Atmos. Environ., 36, 1077–1086, doi:10.1016/S1352-2310(01) 00381-8.
3. City of Shinjuku, 2018.: available at: [http://www.city.shinjuku.lg.jp](http://www.city.shinjuku.lg.jp/), last access: 16 December 2018.
4. Tokyo Metropolitan Government Bureau of Environment, 2018.

http://www.kankyo.metro.tokyo.jp/air/air_pollution/torikumi/result_measurement.html (last access: 16 December 2018).

1. Paatero, P.; Tapper, U. Positive matrix factorization: A non‐negative factor model with optimal utilization of error estimates of data values, *Environ.* **1994**. 5, 111-126, doi: 10.1002/env.3170050203.
2. Norris, G. A., Duvall, R., Brown, S. G., Bai, S., 2014. EPA Positive Matrix Factorization (PMF) 5.0 fundamentals and User Guide Prepared for the US Environmental Protection Agency Office of Research and Development, Washington, DC. Inc., Petaluma.
3. Ulbrich, I. M., Canagaratna, M. R., Zhang, Q., Worsnop, D. R., Jimenez, J. L., 2009. Interpretation of organic components from Positive Matrix Factorization of aerosol mass spectrometric data, Atmos. Chem. Phys., 9(9), 2891-2918, doi: 10.5194/acp-9-2891-2009,
4. Bhanuprasad, S.G., Venkataraman, C., Bhushan, M., 2007. Positive matrix factorization and trajectory modelling for source identification: A new look at Indian Ocean Experiment ship observations, Atmos. Environ., 42: 4836–4852, doi: 10.1016/j.atmosenv.2008.02.041.
5. Callén, M.S., López, J.M., Iturmendi, A., Mastral, A.M., 2013. Nature and sources of particle associated polycyclic aromatic hydrocarbons (PAH) in the atmospheric environment of an urban area, Environ. Pollut., 183: 166–174, doi: 10.1016/j.envpol.2012.11.009.
6. Ma, W. L., Sun, D. Z., Shen, W. G., Yang, M., Qi, H., Liu, L. Y., Shen, J. M., Li, Y. F., 2011. Atmospheric concentrations, sources and gas-particle partitioning of PAHs in Beijing after the 29th Olympic Games, Environ. Pollut., 159, 1794-1801, doi: 10.1016/j.envpol.2011.03.025.
7. Pio, C., Mirante, F., Oliveira, C., Matos, M., Caseiro, A., Oliveira, C., Querol, X., Alves, C., Martins, N., Cerqueira, M., Camões, F., Silva, H. Plana, F., 2013. Size-segregated chemical composition of aerosol emissions in an urban road tunnel in Portugal, Atmos. Environ., 71, 15–25, doi: 10.1016/j.atmosenv.2013.01.037.
8. Harrison, R.M., Jones, A.M., Gietl, J., Yin, J. Green, D.C., 2012. Estimation of the contributions of brake dust, tire wear, and resuspension to nonexhaust traffic particles derived from atmospheric measurements, Environ. Sci. Technol., 46, 6523–6529, doi: 10.1021/es300894r.
9. Zheng, Z., Tang, X., Asa-Awuku, A., Jung, H. S., 2010. Characterization of a method for aerosol generation from heavy fuel oil (HFO) as an alternative to emissions from ship diesel engines, J. Aerosol. Sci., 41, 1143-1151, doi: 10.1016/j.jaerosci.2010.10.002.
10. Konishi, T., Yonemochi, S., Murata, M., 2018. Chemical Composition and Sources Estimation of PM2.5 and Submicron Particles (PM1) in Urban Atmosphere Based on Chemical Components, BUNSEKI KAGAKU, 67, 363-368,doi: 10.2116/analsci.announce1806.
11. Kobayashi, S., Kondo, Y., Fushimi, A., Fujitani, Y., Saito, K., Takami, A., Tanabe, K., 2012. Particulate Matter Emissions from Direct Injection Gasoline Passenger Car, Transactions of Society of Automotive Engineers of Japan., 43, 1009-1014, 10.11351/jsaeronbun.43.1009.
12. Liu, J., Man, R., Ma, S., Li, J., Wu, Q., Peng, J., 2015. Atmospheric levels and health risk of polycyclic aromatic hydrocarbons (PAHs) bound to PM_2.5_ in Guangzhou, China, Mar. Pollut. Bull., 100, 134-143, doi: 10.1016/j.marpolbul.2015.09.014.
13. Zechmeister, H.G., Dullinger, S., Hohenwallner, D., Riss, A., Hanus-Illnar, A. Scharf, S., 2006. Pilot study on road traffic emissions (PAHs, heavy metals) measured by using mosses in a tunnel experiment in Vienna, Austria, Environ. Sci. Pollut. Res. Int., 13, 398–405, doi: 10.1065/espr2006.01.292.
14. Wang, C., Wu, S., Zhou, S., Wang, H., Li, B., Chen, H., Yu, Y Shi, Y., 2015. Polycyclic aromatic hydrocarbons in soils from urban to rural areas in Nanjing: concentration, source, spatial distribution, and potential human health risk, Sci. Total. Environ., 527, 375-383, doi: 10.1016/j.scitotenv.2015.05.025.
15. Wang, K., Shen, Y., Zhang, S., Ye, Y., Shen, Q., Hu, J., Wang, X., 2009. Application of spatial analysis and multivariate analysis techniques in distribution and source study of polycyclic aromatic hydrocarbons in the topsoil of Beijing, China, Environ. Geol., 56, 1041-1050, doi: 10.1007/s00254-008-1204-5.
16. Liu, W., Hopke, P. K., Han, Y., Yi, S., Holsen, T. M., Cybart, S., Kozlowski, K., Milligan, M., 2003. Application of receptor modeling to atmospheric constituents at Potsdam and Stockton, NY, Atmos. Environ., 37, 4997-5007, doi: 10.1016/j.atmosenv.2003.08.036.
17. Masclet, P., Mouvier, G., Nikolaus, K., 1986. Relative decay index and sources of polycyclic aromatic hydrocarbons, Atmos. Environ., 20, 439-446, doi: 10.1016/0004-6981(86)90083-1.
18. Wang, K., Shen, Y., Zhang, S., Ye, Y., Shen, Q., Hu, J., Wang, X., 2009. Application of spatial analysis and multivariate analysis techniques in distribution and source study of polycyclic aromatic hydrocarbons in the topsoil of Beijing, China, Environ. Geol., 56, 1041-1050, doi: 10.1007/s00254-008-1204-5.
19. Lee, J. H., Gigliotti, C. L., Offenberg, J. H., Eisenreich, S. J., Turpin, B. J., 2004. Sources of polycyclic aromatic hydrocarbons to the Hudson River Airshed, Atmos. Environ., 38, 5971-5981, doi: 10.1016/j.atmosenv.2004.07.004.
20. Simoneit, B. R., Medeiros, P. M., Didyk, B. M., 2005. Combustion products of plastics as indicators for refuse burning in the atmosphere, Environ. Sci. Technol., 39, 6961-6970, doi: 10.1021/es050767x.
21. Okuda, T., Okamoto, K., Tanaka, S., Shen, Z., Han, Y., Huo, Z., 2010. Measurement and source identification of polycyclic aromatic hydrocarbons (PAHs) in the aerosol in Xi'an, China, by using automated column chromatography and applying positive matrix factorization (PMF), Sci. Total. Environ., 408, 1909-1914, doi: 10.1016/j.scitotenv.2010.01.040.
22. Cooper, D. A., 2003. Exhaust emissions from ships at berth, Atmos. Environ., 37, 3817-3830, doi: 10.1016/S1352-2310(03)00446-1.
23. Ravindra, K., Sokhi, R., and Van Grieken, R., 2008. Atmospheric polycyclic aromatic hydrocarbons: source attribution, emission factors and regulation, Atmos. Environ., 42, 2895-2921, doi: 10.1016/j.atmosenv.2007.12.010.
24. Hedberg, E., Kristensson, A., Ohlsson, M., Johansson, C., Johansson, P. Å., Swietlicki, E., Vesely, V., Wideqvist, U., Westerholm, R., 2002. Chemical and physical characterization of emissions from birch wood combustion in a wood stove, Atmos. Environ., 36(30), 4823-4837, doi: 10.1016/S1352-2310(02)00417-X.
25. Tokyo Metropolitan Government Bureau of Environment, 2011. http://www.kankyo.metro.tokyo.jp/air/air_pollution/torikumi/pm2_5/pm25-report2011.files/013_inbentori.pdf (last access: 16 December 2018).
26. Gietl, J. K., Lawrence, R., Thorpe, A. J., Harrison, R. M., 2010. Identification of brake wear particles and derivation of a quantitative tracer for brake dust at a major road, Atmos. Environ., 44(2), 141-146, doi: 10.1016/j.atmosenv.2009.10.016.
27. Harrison, R.M., Jones, A.M., Gietl, J., Yin, J. Green, D.C., 2012. Estimation of the contributions of brake dust, tire wear, and resuspension to nonexhaust traffic particles derived from atmospheric measurements. Environ. Sci. Technol., 46, 6523–6529, doi: 10.1021/es300894r.
28. Miguel, A. H., Kirchstetter, T. W., Harley, R. A., 1998. On-road emissions of particulate polycyclic aromatic hydrocarbons and black carbon from gasoline and diesel vehicles, Environ. Sci. Technol., 32, 450-455, doi: 10.1021/es970566w.
29. Zechmeister, H.G., Dullinger, S., Hohenwallner, D., Riss, A., Hanus-Illnar, A. Scharf, S., 2006. Pilot study on road traffic emissions (PAHs, heavy metals) measured by using mosses in a tunnel experiment in Vienna, Austria, Environ. Sci. Pollut. Res. Int., 13, 398–405, doi: 10.1065/espr2006.01.292.
30. Sakata, M., and Marumoto K., 2002. Formation of atmospheric particulate mercury in the Tokyo metropolitan area, Atmos. Environ., 36, 239-246, doi: 10.1016/S1352-2310(01)00432-0.
31. Healy, R. M., O’Connor, I. P., Hellebust, S., Allanic, A., Sodeau, J. R., Wenger, J. C., 2009. Characterisation of single particle from in-port ship emissions, Atmos. Environ., 43: 6408-6414, doi: 10.1016/j.atmosenv.2009.07.039.
32. Tian, H., Cheng, K., Wang, Y., Zhao, D., Lu, L., Jia, W., Hao, J., 2012. Temporal and spatial variation characteristics of atmospheric emissions of Cd, Cr, and Pb from coal in China, Atmos. Environ., 50, 157-163, doi: 10.1016/j.atmosenv.2011.12.045.
33. Mastalerz, M., Hower, J. C., Drobniak, A., Mardon, S. M., Lis, G., 2004. From in-situ coal to fly ash: a study of coal mines and power plants from Indiana, Int. J. Coal. Geol., 59(3-4), 171-192, doi: 10.1016/j.coal.2004.01.005.
34. Han, S., Lee, J. Y., Heo, J., and Kim, Y. P.: Temporal Trend of the Major Contributors for the Particulate Polycyclic Aromatic Hydrocarbons (PAHs) in Seoul, Aerosol Air Qual. Res., doi: 10.4209/aaqr.2018.06.0231, 2018.
